# Development of a Prognostic Model for Oral Cancer by Incorporating Novel Nodal Parameters Beyond Conventional TNM Staging

**DOI:** 10.3390/diagnostics15243133

**Published:** 2025-12-08

**Authors:** Ping-Chia Cheng, Chih-Ming Chang, Li-Jen Liao, Po-Wen Cheng, Wu-Chia Lo

**Affiliations:** 1 Department of Otolaryngology Head and Neck Surgery, Far Eastern Memorial Hospital, New Taipei City 220216, Taiwan; i.cruising@gmail.com (P.-C.C.); b88401077@ntu.edu.tw (C.-M.C.); deniro@mail2000.com.tw (L.-J.L.); powenjapan@yahoo.com.tw (P.-W.C.); 2 Head and Neck Cancer Surveillance and Research Study Group, Far Eastern Memorial Hospital, New Taipei City 220216, Taiwan; 3 Department of Biomedical Engineering, National Yang-Ming University, Taipei 112304, Taiwan; 4 Department of Electrical Engineering, Yuan Ze University, Taoyuan 320315, Taiwan; 5 Graduate Institute of Medicine, Yuan Ze University, Taoyuan 320315, Taiwan

**Keywords:** oral cancer, prognosis, lymph node yield (LNY), lymph node metastases (LNM), lymph node ratio (LNR)

## Abstract

**Background:** Oral cancer is a major global health burden with heterogeneous survival outcomes. This study aimed to identify clinicopathological factors, particularly lymph node-related parameters, associated with prognosis in patients with oral cancer and to construct a survival model for predicting overall survival (OS). **Methods:** A total of 174 patients with oral cancer who underwent surgery between January 2018 and November 2021 were retrospectively analyzed. Clinicopathological variables, including age, gender, body mass index (BMI), pathological T, N and overall stage, tumor subsite, perineural invasion (PNI), lymphovascular invasion (LVI), surgical margin status, lymph node yield (LNY), lymph node metastases (LNM), and lymph node ratio (LNR), were evaluated. Univariate and multivariate Cox regression analyses were performed to identify independent prognostic factors for OS and disease-specific survival (DSS). **Results:** Univariate analysis showed that older age, lower BMI, advanced pathological stage, presence of PNI or LVI, positive/close margins, LNY < 15, LNM ≥ 3, and LNR ≥ 0.0454 were significantly associated with poorer OS. Multivariate analysis identified age ≥ 63 years, pathological stage 3–4, LNY < 15, LNM ≥ 3, and LNR ≥ 0.0454 as independent predictors of OS. LNR ≥ 0.0454 was the only independent predictor of DSS. A survival model incorporating age, pathological stage, LNY, LNM, and LNR demonstrated good discriminatory ability for OS. **Conclusions:** Multiple independent prognostic factors for oral cancer survival were identified. The proposed survival model provides a practical tool for risk stratification and may assist personalized treatment planning, with particular emphasis on lymph node-related parameters.

## 1. Introduction

Oral cancer is a major global health issue, ranking as the sixth most common cancer worldwide with over 600,000 new cases diagnosed annually [1,2]. Despite advances in diagnostic methods, surgical techniques, and multimodal treatments over recent decades, the prognosis remains suboptimal, with 5-year survival rates generally between 60% and 70% in many regions [2,3]. This highlights the need for improved prognostic tools and personalized treatment strategies.

The tumor-node-metastasis (TNM) staging system, established by the American Joint Committee on Cancer (AJCC), has been the cornerstone for oral cancer prognosis [4]. However, patients with identical TNM stages often experience diverse clinical outcomes, indicating that factors beyond tumor size, nodal involvement, and metastasis influence prognosis [5,6]. This has driven research into additional clinicopathological factors as potential prognostic markers. Among these, pathological features such as perineural invasion (PNI), lymphovascular invasion (LVI) and surgical margins have emerged as important prognostic factors. PNI, defined as tumor invasion into nerve sheaths, correlates with higher rates of locoregional recurrence and reduced survival [7,8]. LVI, defined as the presence of tumor cells within lymphatic or blood vessels, has been linked to higher rates of nodal metastasis and poorer outcomes [7,8]. Surgical margin status reflects the completeness of tumor excision, with positive or close margins linked to elevated risks of local recurrence and decreased survival [9].

Recently, lymph node-related parameters have attracted growing attention as valuable prognostic indicators. The lymph node ratio (LNR), defined as the ratio of positive lymph nodes to the total number of lymph nodes examined, has demonstrated significant prognostic value across various head and neck cancers, including oral cavity malignancies [10,11,12]. Ebrahimi et al. reported that LNR independently predicted survival in oral squamous cell carcinoma patients, even after adjusting for traditional TNM staging [10]. Similarly, Patel et al., in a large international study, found that LNR outperformed the conventional nodal staging system in predicting outcomes [11]. The concept of lymph node yield (LNY), representing the total number of lymph nodes retrieved during neck dissection, has also emerged as a potential prognostic factor [13,14]. Ebrahimi et al. demonstrated that higher LNY was associated with improved survival in oral cancer patients, suggesting that more comprehensive lymphadenectomy may have therapeutic benefits [13]. This finding was corroborated by Divi et al., who reported that lymph node count independently predicted mortality in head and neck cancer patients [14]. Furthermore, the absolute number of lymph node metastases (LNM) has also been identified as a strong prognostic indicator, with studies by Roberts et al. suggesting that LNM may be superior to both LNR and conventional AJCC N staging in predicting outcomes [15].

Prognostic nomograms that integrate clinicopathological variables have emerged as valuable tools for personalized risk stratification in oral cancer [16,17,18]. Montero et al. developed a preoperative nomogram incorporating age, sex, clinical T/N stage, and tumor site, enabling individualized risk assessment before surgery [17]. Similarly, Wang et al. constructed postoperative nomograms integrating age, sex, pathological T/N stage, and tumor site to estimate both overall survival (OS) and disease-specific survival (DSS) [18]. These tools may facilitate more informed clinical decision-making regarding treatment intensity, adjuvant therapy, and surveillance strategies. However, both models primarily rely on conventional predictors, lacking integration of important pathological features such as surgical margin status, PNI, LVI, and detailed lymph node-related parameters. Comprehensive analyses incorporating a wider array of clinicopathological features, particularly lymph node-related parameters, remain limited in the literature.

This study aims to identify clinicopathological factors, particularly comprehensive lymph node parameters, associated with OS and DSS in oral cancer patients, and to develop an integrated prognostic survival model for improved risk stratification and personalized treatment planning.

## 2. Materials and Methods

### 2.1. Ethical Considerations

This retrospective study was conducted at a tertiary medical center and approved by the institutional ethical review board (No.113210-E), and the requirement for informed consent was waived due to the retrospective nature of the study. The study adhered to the Declaration of Helsinki.

### 2.2. Study Population and Data Collection

This retrospective study included 174 patients diagnosed with oral cancer who underwent surgery between January 2018 and November 2021. Patient information was extracted from the hospital’s cancer registry database. The collected clinicopathological variables included age, gender, body height (BH), body weight (BW), body mass index (BMI), smoking and alcohol history, tumor size, pathological TNM stage (T stage, N stage, overall stage), tumor subsite, adjuvant treatment, follow-up duration, recurrence status, perineural invasion (PNI), lymphovascular invasion (LVI), surgical margin status, lymph node yield (LNY), lymph node metastases (LNM), lymph node ratio (LNR), and survival status. Adjuvant therapy was classified as radiotherapy (RT), chemotherapy (CT), or concurrent chemoradiotherapy (CRT). The follow-up duration was calculated from the date of cancer diagnosis to either the date of death or the last documented clinical contact. The surveillance protocol consisted of monthly clinical evaluations during the first year, followed by assessments every two months in the second year and every three months until the fifth year. Imaging studies with CT or MRI were performed every six months during the first two years and thereafter annually or as clinically indicated. Patterns of failure were classified as locoregional recurrence or distant metastasis.

### 2.3. Definitions and Cut-Off Values

Pathological T stage, N stage, and overall stage (pStage) were classified according to the 8th edition of the American Joint Committee on Cancer (AJCC) TNM staging system. PNI and LVI were classified as absent or present. Surgical margin was classified as free or positive/close. Cut-off values for key variables were determined by ROC analysis using the Youden index: age (63 years), BMI (23.5 kg/m^2^), overall stage (pStage 1+2 vs. 3+4), LNY (15 nodes), LNM (3 nodes), and LNR (0.0454).

### 2.4. Statistical Analysis

All statistical analyses were performed using STATA version 14.0 (Stata Corporation, College Station, TX, USA). Continuous variables were reported as mean ± standard deviation (interquartile range), while categorical variables were presented as counts and percentages. Survival analysis employed the Kaplan–Meier method, with group differences assessed via the log-rank test. Univariate and multivariate Cox regression analyses were used to identify independent prognostic factors for overall survival (OS) and disease-specific survival (DSS). A prognostic survival model using nomogram was created based on the multivariate analysis, and patients were stratified into three risk groups using a two-threshold approach derived from ROC curve analysis. This dual-threshold method provides clearer differentiation of risk levels compared to traditional single-threshold classification, facilitating more clinically meaningful risk categorization and decision-making. Statistical significance was defined as *p*-value < 0.05.

## 3. Results

The cohort comprised 154 men (88.51%) and 20 women (11.49%), with a mean age of 55.6 ± 9.9 years (Table 1). The mean BMI was 25.5 ± 4.6 kg/m^2^ (range: 15.01–41.15 kg/m^2^). Smoking and alcohol consumption were reported in 135 (77.59%) and 110 (63.22%) patients, respectively. Tumor characteristics were as follows: 40 (22.99%) patients had pT1, 39 (22.41%) had pT2, 45 (25.86%) had pT3, and 50 (28.74%) had pT4 disease. The mean tumor size was 31.5 ± 18.2 mm (range: 1–100 mm). Primary subsites included the tongue (*n* = 64), buccal mucosa (*n* = 55), gingiva (*n* = 30), lip (*n* = 9), retromolar trigone (*n* = 8), floor of mouth (*n* = 5), and hard palate (*n* = 3). Nodal status showed 113 (65.94%) patients with pN0, 19 (10.92%) with pN1, 16 (9.19%) with pN2, and 26 (14.94%) with pN3 disease. Pathological stage distribution was: 34 (19.54%) pStage 1, 25 (14.37%) pStage 2, 38 (21.84%) pStage 3, and 77 (44.25%) pStage 4. Perineural invasion (PNI) and lymphovascular invasion (LVI) were present in 72 (41.38%) and 84 (48.28%) patients, respectively. Surgical margins were clear in 154 (88.51%) patients, and positive or close in 20 (11.49%). The mean surgical margin distance was 5.74 ± 5.60 mm. The mean lymph node yield (LNY) was 28.4 ± 17.6 nodes (range: 1–90), and mean lymph node metastases (LNM) was 1 ± 2.2 nodes (range: 0–15). The mean lymph node ratio (LNR) was 0.428 ± 0.102 (range: 0–0.75), with 41 (23.56%) patients having an LNR ≥ 0.0454.

**Figure 1:**
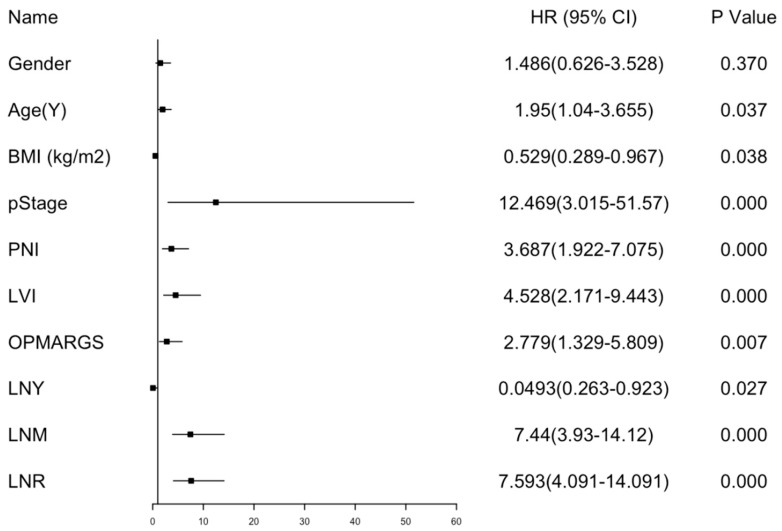
Forest plot of OS with the variables included in the univariable analysis. Note. Abbreviation: OS, overall survival; HR, hazard ratio; 95% CI, 95% confidence interval; BMI, body mass index; pStage, pathological overall stage; PNI, perineural invasion; LVI, lymphovascular invasion; OPMARGS, Surgical margin; LNY, lymph node yield; LNM, lymph node metastases; LNR, lymph node ratio.

At analysis, 131 (75.29%) patients were alive, and 43 (24.71%) had died. The follow-up duration was 869 ± 4454 days. Locoregional recurrence occurred in 23 patients, while 17 patients developed distant metastases. Univariate Cox regression analysis (Table 2 and Figure 1) identified significant factors affecting OS: age ≥ 63 years (HR = 1.950, 95% CI: 1.040–3.655, *p* = 0.037), BMI < 23.5 kg/m^2^ (HR = 0.529, 95%CI: 0.289–0.967, *p* = 0.038), advanced pT stage (pT2: HR = 5.198, 95%CI: 1.139–23.724, *p* = 0.033; pT3: HR = 7.311, 95%CI: 1.672–31.976, *p* = 0.008; pT4: HR = 7.344, 95%CI: 1.688–31.953, *p* = 0.008), advanced pN stage (pN2: HR = 6.291, 95%CI: 2.718–14.564, *p* < 0.001; pN3: HR = 7.457, 95%CI: 3.620–15.362, *p* < 0.001), pStage 3+4 (HR = 12.469, 95%CI: 3.015–51.570, *p* < 0.001), presence of PNI (HR = 3.687, 95%CI: 1.922–7.075, *p* < 0.001), presence of LVI (HR = 4.528, 95%CI: 2.171–9.443, *p* < 0.001), positive surgical margins (HR = 2.779, 95%CI: 1.329–5.809, *p* = 0.007), LNY <15 (HR = 0.049, 95%CI: 0.263–0.923, *p* = 0.027), LNM ≥3 (HR = 7.440, 95%CI: 3.930–14.120, *p* < 0.001), and LNR ≥0.0454 (HR = 7.593, 95%CI: 4.091–14.091, *p* < 0.001). Kaplan–Meier survival analysis revealed significant OS differences by age, pathological stage, LNY, LNM, and LNR. Five-year survival rates were 61% vs. 73% for patients ≥ 63 vs. < 63 years (*p* = 0.037), 96% vs. 58% for pStage 1+2 vs. 3+4 (*p* < 0.001), 75% vs. 51% for LNY ≥ 15 vs. < 15 (*p* = 0.027), 15% vs. 77% for LNM ≥ 3 vs. < 3 (*p* < 0.001), and 23% vs. 85% for LNR ≥ 0.0454 vs. < 0.0454 (*p* < 0.001). Multivariate Cox regression analysis identified the following independent prognostic factors for OS: age ≥63 years (HR = 2.154, 95%CI: 1.115–4.163, *p* = 0.022), pStage 3+4 (HR = 7.424, 95%CI: 1.687–32.666, *p* = 0.008), LNY <15 (HR = 0.355, 95%CI: 0.180–0.698, *p* = 0.003), LNM ≥3 (HR = 3.833, 95%CI: 1.620–9.070, *p* = 0.002), and LNR ≥0.0454 (HR = 2.154, 95%CI: 1.115–4.163, *p* = 0.022).

**Figure 2:**
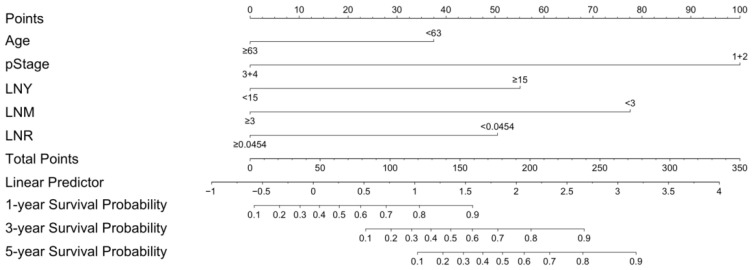
The nomogram for OS in oral cancer patients. Note. Abbreviation: OS, overall survival; pStage, pathological overall stage; LNY, lymph node yield; LNM, lymph node metastases; LNR, lymph node ratio.

**Figure 3:**
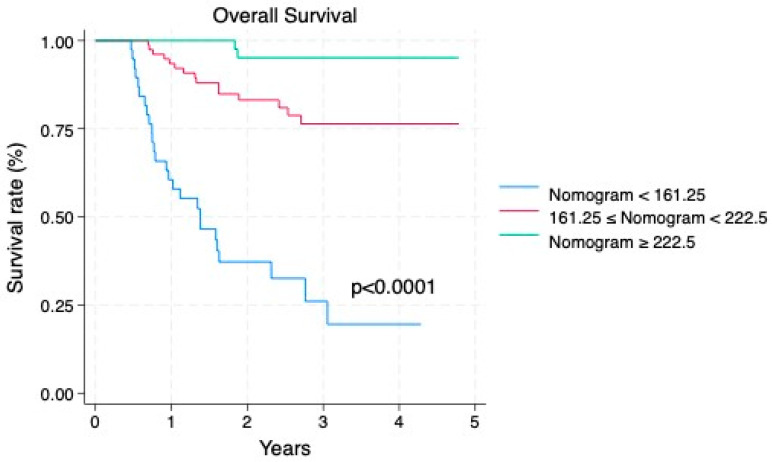
Kaplan–Meier survival curves stratified according to risk groups generated by the survival model. Note. The 1-, 3-, and 5-year overall survival rates were 100.0%, 95.9%, and 95.9% for the low-risk group (nomogram score ≥ 222.5); 93.4%, 76.6%, and 76.6% for the intermediate-risk group (222.5 > score ≥ 161.25); and 60.5%, 26.6%, and 17.8% for the high-risk group (score < 161.25), respectively.

Based on multivariate results, a survival model was constructed to predict OS incorporating age, pathological stage, LNY, LNM, and LNR (Figure 2). ROC curve analysis defined two clinical thresholds of survival model: a low (rule-out) threshold at 161.25 (sensitivity ≥ 90%; observed 90.84%, specificity 60.47%), and a high (rule-in) threshold at 222.5 (specificity ≥ 90%; observed 95.35%, sensitivity 43.51%). Patients were stratified into three risk groups: high-risk (score < 161.25), medium-risk (score 161.25–222.5), and low-risk (score ≥ 222.5). Kaplan–Meier curves showed survival differences among groups (log-rank *p* < 0.001), with 1-, 3-, and 5-year survival rates of 100%, 95.9%, and 95.9% for the low-risk group; 93.4%, 76.6%, and 76.6% for the medium-risk group; and 60.5%, 26.6%, and 17.8% for the high-risk group (Figure 3).

In the univariate analysis for DSS, factors such as advanced nodal stage (pN2 and pN3), pathological stage (pStage 3+4), presence of PNI, presence of LVI, LNM ≥ 3, and LNR ≥ 0.0454 were significantly associated with worse DSS (Table 3). In the multivariate Cox regression analysis, LNR ≥ 0.0454 remained an independent prognostic factor for DSS (HR = 3.986, 95% CI: 1.222–13.002, *p* = 0.025). LVI showed a borderline association (*p* = 0.088), while other variables were not significant after adjustment.

## 4. Discussion

This study comprehensively analyzed various clinicopathological factors associated with survival in patients with oral cancer and developed a prognostic survival model to predict overall survival. Our findings highlight the importance of lymph node-related factors, particularly LNY, LNM, and LNR, as strong predictors of survival in oral cancer patients [10,11,13,14,15]. Based on the identified independent prognostic factors, we developed a survival model, which effectively stratified patients into three prognostic groups with significantly different survival outcomes. Such a tool could be valuable for individualized risk assessment, and may help identify high-risk patients who could benefit from intensified adjuvant therapy or enhanced surveillance. The recognition of lymph node-related factors as robust survival predictors carries important clinical implications. For surgeons, it underscores the necessity of adequate lymph node dissection during neck surgery. For pathologists, it highlights the importance of meticulous lymph node examination and precise reporting. For oncologists, it provides supplementary prognostic information to guide adjuvant treatment decisions.

The demographic and clinical characteristics of our cohort align with previously reported data, with a predominance of male patients and a significant association with smoking and alcohol consumption [2]. The high proportion of advanced-stage disease (66.09% with pStage 3–4) reflects the reality of oral cancer presentation in many regions, where delayed diagnosis remains a challenge [2].

Age emerged as an independent prognostic factor; patients aged ≥63 years exhibited significantly poorer outcomes, consistent with earlier studies linking older age to worse prognosis in oral cancer [19,20]. This may relate to age-associated comorbidities, diminished treatment tolerance, and possibly more aggressive tumor biology.

As anticipated, pathological stage, combining tumor and nodal status, strongly correlated with survival [19]. Patients with advanced stage (pStage 3–4) fared worse than those with early stage (pStage 1–2), underscoring the critical need for early detection and treatment [2,5].

Tumor subsite demonstrated prognostic relevance, with buccal mucosa showing slightly better survival than tongue, although this difference did not reach statistical significance in the univariate analysis. Subsite-stratified lymph node assessments (Supplementary Table S1) revealed comparable LNY, LNM, and LNR among tongue, buccal mucosa, and gingiva/other subsites, indicating consistent surgical quality across anatomical locations.

Tumor subsite showed distinct prognostic patterns, with tongue cancers tending toward poorer survival than buccal mucosa cancers, although this difference did not retain significance in multivariate analysis. Subsite-stratified lymph node assessment (Supplementary Table S1) demonstrated comparable lymph node yields across oral cavity subsites, suggesting consistent neck dissection quality and reducing the likelihood of surgical bias in nodal evaluation. Buccal mucosa cancers were characterized by a lower propensity for nodal dissemination, reflected by reduced rates of LNM and a lower lymph node ratio, whereas tongue and gingiva/other subsites exhibited a higher nodal burden, supporting biologically distinct metastatic behaviors with potential implications for prognosis and subsite-specific risk stratification.

Lymph node-related variables proved to be powerful independent survival predictors. LNY ≥ 15 was associated with improved survival outcomes, suggesting that adequate lymph node sampling during neck dissection may have prognostic implications [13,14]. This finding supports the concept that a more thorough lymphadenectomy may not only improve staging accuracy but may also have therapeutic benefits by removing potential micrometastases [21,22]. Stampe et al. recently affirmed the prognostic significance of LNY as a quality metric in oral cancer neck dissections and survival [23].

LNM was similarly prognostic, with ≥3 metastatic nodes linked to poorer survival, corroborating previous evidence of a threshold effect of nodal burden [15,24]. Recent work by Struckmeier et al. demonstrated that LNY, LNR, and LNM all provide valuable prognostic insights, with LNM showing particularly strong predictive value in oral squamous cell carcinoma [25].

The most compelling finding of our study is the strong prognostic value of LNR. Patients with LNR ≥0.0454 had significantly worse OS and DSS. The prognostic significance of LNR persisted in multivariate analysis, suggesting that it provides additional prognostic information beyond conventional TNM staging. LNR may be particularly valuable as it accounts for both the extent of nodal disease (number of positive nodes) and the adequacy of lymph node evaluation (total number of nodes examined). Our results support LNR as a powerful marker for risk stratification in oral cancer management, with potential to guide surgical strategies, adjuvant therapy decisions, and surveillance intensity. Recent studies by Haraguchi et al. and Mamic et al. further reinforce LNR’s prognostic power across diverse populations [26,27]. Additionally, Verde-Sanchez et al. have proposed a predictive model incorporating lymph node density, demonstrating its significant prognostic value in treatment planning for oral SCC patients [28]. Building upon these concepts, our study provides a more comprehensive assessment of lymph node-related parameters, including LNY, LNM, and LNR, to deliver prognostic insights beyond the TNM framework. This approach captures the combined effects of nodal burden and dissection adequacy.

PNI and LVI were significant prognostic factors in univariate analysis but did not emerge as independent predictors in multivariate analysis for overall survival. These findings suggest that while these histopathological features are important markers of tumor aggressiveness, their prognostic impact may be partially mediated through their association with other factors, particularly nodal status. Furthermore, despite the significance of PNI, LVI in univariate analysis, no one has assessed the state of the organs of immunobiological surveillance with the same other parameters in patients, so it is not possible to consider their value as independent predictors in multivariate analysis for OS. A recent systematic review and meta-analysis by Alqutub et al. confirmed that histopathological features, including PNI and LVI, are indeed important predictors of lymph node metastasis in oral cavity squamous cell carcinoma, though their independent prognostic value varies across studies [8].

Surgical margin status was significantly associated with survival in univariate analysis but not in multivariate analysis. This could be attributed to the relatively small number of patients with positive or close margins (11.5%) in our cohort, which may have limited statistical power. Nevertheless, the importance of achieving negative surgical margins in oral cancer management cannot be overstated, as it remains a fundamental principle of oncologic surgery.

The BMI finding in our univariate analysis (BMI < 23.5 kg/m^2^ associated with worse survival) aligns with emerging evidence suggesting that nutritional status and body composition may influence cancer outcomes. Low BMI may reflect cancer cachexia, malnutrition, or compromised physiological reserves, all of which can adversely affect survival. Conversely, elevated BMI has been associated with chronic inflammatory states and metabolic dysfunction that impair antitumor immune responses [29]. However, it is noteworthy that in our analysis, patients with BMI < 23.5 showed worse outcomes, suggesting that the adverse effects of malnutrition and depletion outweigh potential protective anti-inflammatory effects at lower BMI ranges in this cohort. The complex relationship between BMI, metabolic status, and antitumor immunity warrants further investigation. Regardless, BMI was not significant in multivariate analysis, indicating confounding by stronger prognostic factors.

Overall, our findings and accumulating evidence advocate for comprehensive lymph node assessment, including LNR, LNY, and LNM, as superior prognostic markers compared to conventional nodal staging alone [30]. This supports the need for standardized pathological reporting of lymph node parameters in oral cancer.

Despite these significant findings, our study has several limitations. First, its retrospective nature introduces potential selection bias and limits causal inference. Second, the relatively small sample size and single-institution setting may limit the generalizability of our findings. External validation of the prognostic survival model in an independent cohort is necessary to confirm its predictive accuracy and clinical utility. Future studies should focus on validating these findings in larger, multi-institutional cohorts and integrating molecular and genomic markers with clinicopathological factors to develop more comprehensive prognostic models.

## 5. Conclusions

This research identified several independent prognostic factors associated with survival outcomes. Specifically, age ≥ 63 years, advanced pathological stage (pStage 3+4), LNY < 15, LNM ≥ 3, and LNR ≥ 0.0454 were strong independent predictors of overall survival. These findings emphasize the vital role of comprehensive lymph node assessment in oral cancer management for precise staging and reliable prognostication.

## Figures and Tables

**Table 1:** Clinical and pathological characteristics of included patients.

Characteristics	Characteristic Options	*N* (%) or Mean ± SD (IQR)
Age (y)		55.6 ± 9.9 (33–78)
Gender	Male/female	154 (88.51%)/20 (11.49%)
BH (cm)		165.4 ± 7.7 (135–183)
BW (kg)		70.1 ± 14.3 (36–110)
BMI (kg/m^2^)		25.5 ± 4.6 (15.01–41.15)
Smoking	Yes/No	135 (77.59%)/39 (22.41%)
Alcohol	Yes/No	110 (63.22%)/64 (36.78%)
pT	1/2/3/4	40 (22.99%)/39 (22.41%)/45 (25.86%)/50 (28.74%)
pN	0/1/2/3	113 (65.94%)/19 (10.92%)/16 (9.19%)/26 (14.94%)
pStage	1/2/3/4	34 (19.54%)/25 (14.37%)/38 (21.84%)/77 (44.25%)
Tumor subsite	Tongue	64 (36.78%)
	Buccal mucosa	55 (31.61%)
	Gingiva	30 (17.24%)
	Lip	9 (5.17%)
	Retromolar trigone	8 (4.60%)
	Floor of mouth	5 (2.87%)
	Hard palate	3 (1.72%)
Adjuvant treatment	No/RT/CT/CRT	52 (29.89%)/10 (5.75%)/22 (12.64%)/90 (51.72%)
Follow-up duration (days)		869 ± 4454 (172–1748)
Survival status	Yes/No	131 (75.29%)/43 (24.71%)
Recurrence status	No/Locoregional/Distant	134 (77.01%)/23 (13.21%)/17 (9.77%)
PNI	Absent/Present	102 (58.62%)/72 (41.38%)
LVI	Absent/Present	90 (51.72%)/84 (48.28%)
Tumor size (mm)		31.5 ± 18.2 (1–100)
Surgical margin	Free/Positive or very close	154 (88.51%)/20 (11.5%)
Surgical margin distance (mm)		5.74 ± 5.60 (0.00–23.00)
LNY		28.4 ± 17.6 (1–90)
LNY group	<15/≥15	39 (22.41%)/135 (77.59%)
LNM		1 ± 2.2 (0–15)
LNM group	<3/≥3	154 (88.51%)/20 (11.49%)
LNR		0.428 ± 0.102 (0–0.75)
LNR group	<0.0454/≥0.0454	133 (76.44%)/41 (23.56%)

Abbreviation: *N,* number; SD, standard deviation; IQR, interquartile range; BH, body height; BW, body weight; BMI, body mass index; pT, pathological tumor stage, pN, pathological nodal stage; pStage, pathological overall stage; PNI, perineural invasion; LVI, lymphovascular invasion; LNY, lymph node yield; LNM, lymph node metastases; LNR, lymph node ratio.

**Table 2:** Univariate and multivariate COX regression analyses of factors related to OS in oral cancer patients.

	Univariate				Multivariate (Stepwise)			
	HR	95% CI		*p*-Value	HR	95%CI		*p*-Value
Gender								
Female	Ref							
Male	1.486	0.626	3.528	0.370				
Age(Y)								
<63	Ref				Ref			
≥63	1.950	1.040	3.655	0.037 *	2.154	1.115	4.163	0.022 *
BMI (kg/m^2^)								
<23.5	Ref							
≥23.5	0.529	0.289	0.967	0.038 *				
pT								
1	Ref							
2	5.198	1.139	23.724	0.033 *				
3	7.311	1.672	31.976	0.008 *				
4	7.344	1.688	31.953	0.008 *				
pN								
0	Ref							
1	1.795	0.59	5.455	0.303				
2	6.291	2.718	14.564	<0.001 *				
3	7.457	3.620	15.362	<0.001 *				
pStage								
1+2	Ref				Ref			
3+4	12.469	3.015	51.570	<0.001 *	7.424	1.687	32.666	0.008 *
Tumor subsite								
Tongue	Ref							
Buccal mucosa	0.482	0.218	1.065	0.071				
Gingiva and others	0.900	0.457	1.772	0.760				
PNI								
Absent	Ref							
Present	3.687	1.922	7.075	<0.001 *				
LVI								
Absent	Ref							
Present	4.528	2.171	9.443	<0.001 *				
Surgical margin								
Negative	Ref							
Close/Positive	2.779	1.329	5.809	0.007 *				
LNY								
<15	Ref				Ref			
≥15	0.049	0.263	0.923	0.027 *	0.355	0.180	0.698	0.003 *
LNM								
<3	Ref				Ref			
≥3	7.440	3.930	14.120	<0.001 *	3.833	1.620	9.070	0.002 *
LNR								
<0.0454	Ref				Ref			
≥0.0454	7.593	4.091	14.091	<0.001 *	2.154	1.115	4.163	0.022 *

* Statistical significance with *p* < 0.05 is highlighted with an asterisk and bold font. Abbreviation: HR, hazard ratio; 95% CI, 95% confidence interval; OS, overall survival; BMI, body mass index; pT, pathological tumor stage, pN, pathological nodal stage; pStage, pathological overall stage; PNI, perineural invasion; LVI, lymphovascular invasion; LNY, lymph node yield; LNM, lymph node metastases; LNR, lymph node ratio.

**Table 3:** Univariate and multivariate COX regression analyses of factors related to DSS in oral cancer patients.

	Univariate (Firth Cox)				Multivariate (Stepwise)			
	HR	95%CI		*p*-Value	HR	95%CI		*p*-Value
Gender								
Female	Ref							
Male	1.784	0.548	5.801	0.356				
Age (Y)								
<63	Ref							
≥63	2.004	0.801	5.012	0.144				
BMI (kg/m^2^)								
<23.5	Ref							
≥23.5	0.515	0.213	1.245	0.139				
pT								
1	Ref							
2	3.771	0.568	25.053	NA				
3	4.768	0.758	29.983	NA				
4	4.419	0.702	27.799	0.056				
pN								
0	Ref							
1	3.869	0.948	15.788	0.067				
2	7.703	2.079	28.532	0.004 *				
3	9.038	2.922	27.959	<0.001 *				
pStage								
1+2	Ref							
3+4	23.955	3.305	3046.586	<0.001 *				
Tumor subsite								
Tongue	Ref							
Buccal mucosa	0.312	0.086	1.135	0.077				
Gingiva and other	0.798	0.304	2.098	0.648				
PNI								
Absent	Ref							
Present	4.402	1.623	11.935	0.001 *				
LVI								
Absent	Ref							
Present	5.963	1.839	19.340	<0.001 *	2.895	0.774	10.823	0.088
Surgical margin								
Negative	Ref							
Close/Positive	2.728	0.934	7.969	0.089				
LNY								
<15	Ref							
≥15	0.480	0.192	1.198	0.123				
LNM								
<3	Ref							
≥3	8.195	3.315	20.260	<0.001 *	1.705	0.548	5.306	0.330
LNR								
<0.0454	Ref							
≥0.0454	8.048	3.205	20.208	<0.001 *	3.986	1.222	13.002	0.025 *

* Statistical significance with *p* < 0.05 is highlighted with an asterisk and bold font. Abbreviation: HR, hazard ratio; 95% CI, 95% confidence interval; DSS, disease-specific survival; BMI, body mass index; PNI, perineural invasion; LVI, lymphovascular invasion; LNY, lymph node yield; LNM, lymph node metastases; LNR, lymph node ratio.

## Data Availability

The original contributions presented in this study are included in the article/Supplementary Materials. Further inquiries can be directed to the corresponding author. Data available on request due to restrictions related to patient privacy and institutional ethical regulations.

## Supplementary Materials

The following supporting information can be downloaded at: https://www.mdpi.com/article/10.3390/diagnostics15243133/s1, Table S1: Comparison of categorized lymphatic parameters across subsites.

## Abbreviations

The following abbreviations are used in this manuscript:

LNY	Lymph Node Yield
LNM	Lymph Node Metastases
LNR	Lymph Node Ratio
OS	Overall Survival
DSS	Disease-Specific Survival
PNI	Perineural Invasion
LVI	Lymphovascular Invasion
BMI	Body Mass Index
BH	Body Height
BW	Body Weight
TNM	Tumor-Node-Metastasis
AJCC	American Joint Committee on Cancer
pStage	Pathological Stage
HR	Hazard Ratio
CI	Confidence Interval
ROC	Receiver Operating Characteristic
SD	Standard Deviation
IQR	Interquartile Range
