# Supplementary material for: Development of a Prognostic Model for Oral Cancer by Incorporating Novel Nodal Parameters Beyond Conventional TNM Staging
Source: Diagnostics (Basel). 2025 Dec 8;15(24):3133. doi: 10.3390/diagnostics15243133 (PMC12731973; doi:10.3390/diagnostics15243133)
Supplement: Supplementary file 1 [file diagnostics-15-03133-s001.zip › diagnostics-4013776-supplementary.pdf]

Supplementary Table S1. Comparison of categorized lymphatic parameters across subsites

	Tongue	Buccal mucosa	Gingiva & Others	p-value (χ^2)
LNY				
<15	15 (22.34%)	15 (27.27%)	9 (16.36%)	0.379
≥15	49 (76.56%)	40 (72.73%)	46 (77.59%)	
LNM				
<3	54 (84.38%)	52 (94.55%)	48 (87.27%)	0.209
≥3	10 (15.62%)	3 (5.45%)	7 (12.73%)	
LNR				
< 0.0454	44 (68.75%)	47 (85.45%)	42 (76.36%)	0.101
≥0.0454	20 (31.25%)	8 (14.55%)	13 (23.64%)	

*Statistical significance with $p < 0.05$ is highlighted with an asterisk and bold font. Abbreviation: LNY, lymph node yield; LNM, lymph node metastases; LNR, lymph node ratio.
